# 250 Gb/s All-Optical XNOR Logic Using a Single QD-SOA-MZI: Demonstration and Comprehensive Performance Analysis

**DOI:** 10.3390/mi17040441

**Published:** 2026-04-01

**Authors:** Amer Kotb, Bisheng Zhu, Jiali Cui, Kyriakos E. Zoiros

**Affiliations:** 1 School of Chips, XJTLU Entrepreneur College (Taicang), Xi’an Jiaotong-Liverpool University, Taicang 215400, China; bisheng.zhu22@student.xjtlu.edu.cn (B.Z.); jiali.cui25@student.xjtlu.edu.cn (J.C.); 2 Department of Physics, Faculty of Science, Fayoum University, Fayoum 63514, Egypt; 3 Department of Electrical Engineering and Electronics, University of Liverpool, Liverpool L69 3GJ, UK; 4 Lightwave Communications Research Group, Department of Electrical and Computer Engineering, School of Engineering, Democritus University of Thrace, 67100 Xanthi, Greece

**Keywords:** exclusive-NOR (XNOR), quantum-dot semiconductor optical amplifier (QD-SOA), Mach–Zehnder interferometer (MZI), cross-phase modulation (XPM), quality factor (QF)

## Abstract

Increasing data rates in optical networks require ultra-fast all-optical logic gates to avoid electro-optic conversion bottlenecks. This work presents a numerical simulation and performance analysis of an all-optical XNOR logic gate operating at 250 Gb/s, implemented using a single quantum-dot semiconductor optical amplifier (QD-SOA) embedded in a Mach–Zehnder interferometer (MZI). Using the QD-SOA’s ultrafast carrier dynamics and high nonlinearity, the gate achieves a quality factor (QF) of 26.30 at 250 Gb/s, corresponding to a theoretical bit-error rate below 10^−9^. A systematic numerical investigation examines performance dependence on six critical parameters. Data rate analysis shows that the gate maintains QF > 6 up to 700 Gb/s, with QF = 10.47 at this maximum reliable speed, providing a safety margin of approximately 1.8× above the QF = 6 threshold. Performance degrades progressively thereafter, with QF falling to 5.18 at 800 Gb/s and 0.73 at 1 Tb/s due to finite carrier recovery dynamics. Pulse energy optimization identifies an optimum at 0.20 pJ, beyond which gain saturation and nonlinear effects degrade performance below QF = 6 at 0.40 pJ. Continuous-wave probe power exhibits optimal operation at 0.40 mW, with failure above 0.80 mW. Injection current density analysis establishes an optimal bias at 4 kA/cm^2^, where balanced gain and nonlinearity yield peak performance. Noise tolerance assessment demonstrates operation up to a spontaneous emission factor of 6 and phase noise below 6 × 10^−14^ rad^2^/Hz, beyond which signal integrity collapses. This parameter sweep delineates the operational envelope and optimization guidelines for QD-SOA-MZI-based all-optical logic, confirming its potential as a compact core component for future ultra-high-speed optical communication and signal processing systems.

## 1. Introduction

Global data traffic growth, driven by video streaming, cloud computing, and internet of things (IoT) applications, is approaching the limits of conventional optical communication networks [1,2]. One bottleneck in these systems lies in the optical–electrical–optical (O-E-O) conversion required for fundamental signal processing tasks such as routing, header recognition, and encryption. All-optical signal processing addresses this bottleneck by performing logic operations directly on optical signals, offering potential for higher data rates, lower latency, and reduced power consumption [3,4]. The all-optical logic gate serves as the fundamental building block for complex optical computing, switching, and regeneration systems [5,6,7].

Implementing these gates at speeds exceeding 100 Gb/s demands highly nonlinear, ultrafast, and compact photonic components. Semiconductor optical amplifiers (SOAs) have been extensively studied for this purpose due to their strong nonlinearities, notably cross-gain modulation (XGM) and cross-phase modulation (XPM), their compact footprint, and their potential for monolithic integration with other photonic circuits [8,9,10]. However, conventional bulk or quantum-well (QW) SOAs are intrinsically limited by relatively slow carrier recovery times, typically in the range of tens to hundreds of picoseconds [11,12]. This slow recovery induces significant pattern-dependent effects and inter-symbol interference (ISI), which critically constrain their operational speed in high-bit-rate, interferometric configurations [13,14,15].

Quantum-dot semiconductor optical amplifiers (QD-SOAs) offer advantages over bulk and quantum-well counterparts due to their three-dimensional carrier confinement and discrete density of states [16,17,18,19,20,21]. These include ultrafast carrier dynamics, with sub-picosecond intradot relaxation and picosecond interdot/wet-layer recovery; a broader and flatter gain spectrum; higher saturation output power; and lower noise figure [22,23]. The decoupling of phase recovery from slow carrier-reservoir replenishment enables a rapid nonlinear phase shift [24,25]. These properties allow QD-SOAs to support high-speed operation at 250 Gb/s and beyond with reduced patterning effects, making them suitable for next-generation all-optical processing [26,27,28,29,30].

To efficiently convert the ultrafast phase nonlinearity of a QD-SOA into a clear amplitude-modulated output signal, an interferometric architecture is essential. The Mach–Zehnder interferometer (MZI) is a well-established and highly effective scheme for this purpose [31,32,33,34]. In particular, QD-SOA-based MZIs have emerged as a prominent platform for implementing various all-optical logic gates, including AND, XOR, and NOR, due to their superior high-speed potential compared to bulk-SOA counterparts [35,36,37,38]. In this work, we build upon this established platform by employing an asymmetric MZI with a single QD-SOA placed in one of its arms. This specific configuration offers significant practical benefits: it simplifies the design and control complexity compared to dual-SOA MZI schemes, reducing fabrication challenges and the need for precise dual-bias matching; it effectively exploits the strong and rapid cross-phase modulation (XPM) induced by the input data pulses on a co-propagating continuous-wave (CW) probe beam; and it provides a stable, high-extinction-ratio switching mechanism, translating the induced nonlinear phase shift into a robust amplitude-modulated output at the interferometric port [39].

Within the suite of essential Boolean logic functions, the exclusive-NOR (XNOR) gate is of particular strategic importance. The XNOR operation, which yields a logical ‘1’ only when its two input bits are identical, is a fundamental and versatile function [40,41,42,43,44]. Its applications are critical in advanced optical networks and computing, including but not limited to bit-level pattern matching for address and header recognition, parity generation and checking for optical error detection, arithmetic logic units (ALUs) for optical computing, and fundamental operations in optical encryption and cryptography [40,41,42,45,46,47,48]. Therefore, the realization of a high-speed, high-fidelity all-optical XNOR gate is an important step in route to realizing practical and functional all-optical processors and routers.

A rigorous and quantitative metric is required to evaluate and benchmark the performance of such ultra-high-speed logic gates. The quality factor (QF), a well-established metric derived from the standard QF in digital communications, serves this critical role [49,50,51]. It provides a single, powerful figure of merit that directly quantifies the clarity and integrity of the logic output [52,53]. A higher QF indicates superior separation between the ‘1’ and ‘0’ logic levels, which correlates directly with a lower bit-error rate (BER) and enhanced operational robustness of the gate [52]. Its use enables clear performance comparison across different designs and under varying operational conditions.

In this paper, we propose a model and comprehensively analyze a novel, ultra-high-speed all-optical XNOR gate based on a single QD-SOA within an asymmetric MZI (QD-SOA-MZI) configuration. We successfully demonstrate full XNOR functionality at an ultra-high speed of 250 Gb/s, achieving a quality factor of 26.30, which corresponds to a theoretical bit-error rate well below 10^−9^. Beyond this primary demonstration, we conduct a thorough parametric investigation to establish the operational robustness and practical design guidelines for the proposed gate. This involves a systematic study of the variation in the achieved QF with key operational parameters, including data rate, input pulse energy, CW probe power, injection current density, amplified spontaneous emission (ASE) noise, and phase noise. This work not only demonstrates a high-performance XNOR gate at 250 Gb/s using a simplified and integrable architecture but also provides essential insights for its performance optimization, representing a significant advancement towards the realization of Tb/s-class all-optical signal processing systems [54,55,56,57,58,59,60].

## 2. Operating Principle and Numerical Model

This section details the proposed XNOR gate’s operational principle and the comprehensive numerical framework used for its analysis. First, the unique structural advantages of the QD-SOA as the active nonlinear element are established. Subsequently, the gate architecture and the carrier dynamics model governing its ultrafast response are presented.

### 2.1. Structural and Dynamical Advantages of QD-SOA

The QD-SOA enables 250 Gb/s operation due to three-dimensional quantum confinement of carriers within its active region (AR) semiconductor nanostructures. Unlike bulk or quantum-well materials with continuous energy bands, quantum dots possess a discrete, atomic-like density of states, primarily composed of ground (GS) and excited (ES) energy levels [61]. This discrete structure fundamentally alters the electronic properties, enabling ultrafast carrier dynamics, high differential gain, and low noise.

In practical devices, sufficient modal gain and integrability are achieved through a multi-layer stack of QD layers, typically self-assembled on substrates like GaAs/InGaAs via the Stranski–Krastanov mode. This multilayer design serves critical functions: (i) it enhances the optical confinement factor (Γ) by increasing the overlap between the optical field and the AR; (ii) it compensates for inhomogeneous broadening due to variations in QD size and composition, resulting in a smoother gain spectrum; and (iii) it allows for the engineering of polarization-insensitive gain, enhancing practicality [61,62].

**Figure 1:**
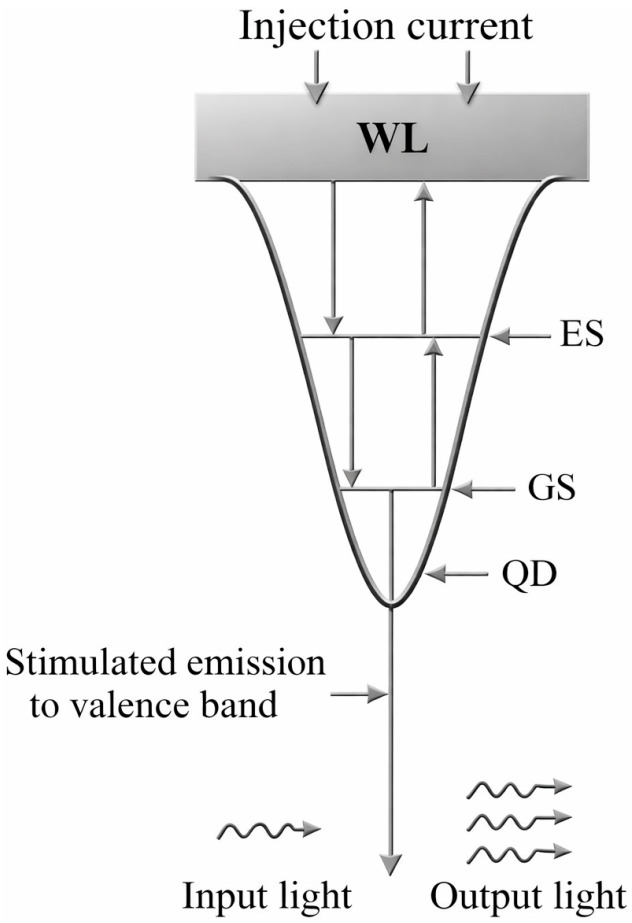
Schematic of the discrete energy levels and carrier relaxation pathways of QD [63], “reprinted with permission from Ref. [63], licensed under CC-BY 4.0”.

Crucially, the QDs, along with the surrounding wetting layer (WL), form an efficient carrier relaxation system. The WL acts as a carrier reservoir. Upon electrical injection, carriers are captured from the WL continuum to the discrete ES on a picosecond timescale (*τ_WL_*_→*ES*_) and subsequently relax to the GS via an ultrafast intradot process (*τ_ES_*_→*GS*_) that can be sub-picosecond. This efficient, hierarchical relaxation path—WL → ES → GS—is the key physical mechanism enabling picosecond-scale gain recovery. It allows the QD-SOA to rapidly replenish carriers depleted by an optical pulse, thereby suppressing patterning effects and supporting ultra-high-speed signal processing far beyond the capabilities of conventional SOAs, as conceptually illustrated in Figure 1 [63].

### 2.2. Gate Architecture and Switching Principle

**Figure 2:**
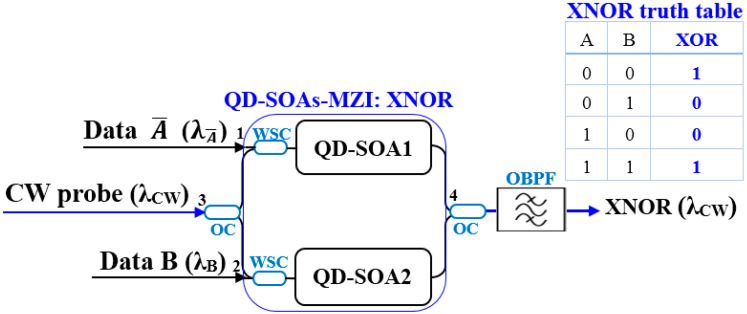
Schematic of the proposed all-optical XNOR gate based on a single QD-SOA-MZI. OC: 3 dB optical coupler. WSC: wavelength-selective coupler. OBPF: optical bandpass filter.

The proposed all-optical XNOR gate, whose schematic is depicted in Figure 2, employs a MZI configuration with a single QD-SOA embedded in one arm.

A continuous-wave (CW) laser beam, serving as the probe signal at wavelength *λ_CW_*, is injected into the input port of the MZI and equally divided by the first 3 dB optical coupler (OC). The two resulting probe components propagate through the upper and lower interferometer arms. Crucially, the logical data signals designated for the XNOR operation are introduced asymmetrically. In this design, the input signals are the logical complement $\bar{A}$ and the signal *B*. The $\bar{A}$ signal is obtained by passing the original data stream A through an all-optical NOT gate, which can be implemented using a separate SOA-based configuration operating in cross-gain modulation (XGM) mode [35,38]. These data streams, at wavelengths *λ_Ā_* and *λ_B_*, respectively, are combined with the CW probe and co-injected into the single QD-SOA arm via wavelength-selective couplers (WSCs). The WSCs ensure efficient coupling of the data while allowing the probe to pass through the QD-SOA with minimal loss, playing a critical role in wavelength-division multiplexing (WDM) of the signals within the gate.

The data pulses $\bar{A}$ and *B* act as pumps that dynamically modulate the carrier density within the QD-SOA via stimulated emission. This carrier depletion perturbs the complex refractive index of the active region, inducing a nonlinear phase shift Δ*ϕ*(*t*) specifically on the probe component co-propagating in the same arm through the XPM effect. The probe component traversing the complementary, SOA-free reference arm experiences no such phase perturbation.

The logical operation is realized based on the phase relationship of the recombining probe beams at the second 3 dBOC, which dictates the nature of their interference at output port 4. The condition for destructive or constructive interference is governed by the combined state of the input data:

- When $\bar{A}$ and *B* are identical (both ‘0’ or both ‘1’), the induced carrier dynamics leave the phase balance of the MZI relatively undisturbed (Δ*ϕ* ≈ 0). The two probe components thus recombine destructively at output port 4, resulting in a low-amplitude output signal interpreted as logic ‘0’.
- When $\bar{A}$ and *B* differ ($\bar{A}$ = 0, *B* = 1) or ($\bar{A}$ = 1, *B* = 0), the pronounced carrier depletion in the QD-SOA arm disrupts the MZI’s symmetry, imparting a significant phase shift (Δ*ϕ* ≈ *π*) to the probe. This phase difference leads to constructive interference at the output port, yielding a high-amplitude signal corresponding to logic ‘1’.

Thus, the interference condition at port 4, dictated by the XPM-induced phase shift, maps the combined logical state of inputs A and B onto the amplitude of the probe beam, physically implementing the XNOR operation (A ⊙ B = ($\bar{A}$ ⊕ B)). The correspondence between input states and output power levels follows the XNOR truth table provided in Figure 2. To complete the gate operation, an optical bandpass filter (OBPF) extracts the modulated probe at *λ_CW_* while rejecting the original data wavelengths, yielding a clean, wavelength-converted logic output.

The wavelength values used in this simulation (*λ_A_* = *λ_B_* = 1580 nm for data signals, *λ_CW_* = 1539 nm for probe) are selected based on the gain bandwidth of InAs/GaAs QD-SOAs (~100 nm spanning 1500–1600 nm) [61,63] and the need for sufficient spectral separation (41 nm) to enable rejection of data wavelengths by the output optical bandpass filter. The 1539 nm probe wavelength aligns with ITU-T grid channel 34, facilitating integration with standard WDM infrastructure, while the 1580 nm data signals occupy the L-band. For experimental realization, a narrow-linewidth (<100 kHz) DFB laser diode for the CW probe is recommended, such as Thorlabs SFL1550P (tunable to 1539 nm), Emcore 1782 (1539 nm ITU channel), or Pure Photonics PPCL300 (channel-selectable). For 250 Gb/s data signals at 1580 nm, suitable sources include mode-locked laser diodes (e.g., Calmar Laser PSL-10 tunable 1560–1610 nm, <0.3 ps pulses) or externally modulated CW lasers using LiNbO_3_ modulators driven by high-speed pattern generators. For applications requiring strict adherence to the 1550 nm C-band, the scheme can be shifted to 1550 nm data with 1510 nm or 1590 nm probe, provided the QD-SOA gain spectrum supports these wavelengths.

### 2.3. Numerical Model for QD-SOA Dynamics

The high-speed performance of the proposed XNOR gate is rigorously simulated using a comprehensive numerical model that accounts for the unique energy level structure and ultrafast nonlinear effects in the QD-SOA [64,65,66,67]. The model goes beyond standard rate equations by explicitly including the time-dependent gain dynamics resulting from both interband and intraband processes, such as carrier heating (CH) and spectral hole burning (SHB). These effects are critical for accurately simulating the device’s response to 250 Gb/s pulses.

The evolution of the QD-SOA’s gain is described by a set of coupled differential equations that model the interactions between the QD ground state and the WL, as well as the ultrafast nonlinearities. The time-dependent power gain for the QD-SOA, integrated over its longitudinal dimension z ∈ [0, L], is governed by [5,68]:

#### 2.3.1. Carrier Dynamics Between QD States and WL

The gain recovery of the QD ground state, represented by *h_d_*(*t*), is driven by carrier capture from the WL and depleted by stimulated emission and recombination:

(1) $$\frac{dh_{d}(t)}{dt}=\frac{h_{w}(t)}{\tau_{dw}}(1-\frac{h_{d}(t)}{h_{0}})-\frac{h_{d}(t)}{\tau_{dr}}-\left(exp\left[h_{d}(t)+h_{CH}(t)+h_{SHB}(t)\right]-1\right)\;\frac{P_{in,QDSOA}(t)}{E_{sat}}$$

Here, *h_d_*(*t*) is the integrated logarithmic gain contribution from the QD ground state. The first term models the carrier capture from the WL, where *h_w_*(*t*) is the WL gain contribution and *τ_dw_* is the characteristic capture time. The term (1 − *h_d_*(*t*)*/h*_0_) represents the Pauli blocking factor, accounting for state filling. The second term describes the loss of carriers due to spontaneous recombination with a time constant τ_dr_. The final term accounts for gain saturation due to stimulated emission, where *P_in,QD-SOA_*(*t*) is the instantaneous input optical power and *E_sat_* is the saturation energy.

#### 2.3.2. WL Carrier Dynamics

The carrier density in the WL, related to *h_w_*(*t*), is governed by electrical injection, recombination, and carrier exchange with the QDs:

(2) $$\frac{dh_{w}(t)}{dt}=\frac{h_{in}}{\tau_{wr}}(1-\frac{h_{w}(t)}{h_{0}})-\frac{h_{w}(t)}{\tau_{wr}}-\frac{h_{w}(t)}{\tau_{wd}}(1-\frac{h_{d}(t)}{h_{0}})$$

In this equation, the first term represents the electrical pumping, where *h_in_* is a parameter proportional to the injection current density *J*. The term (1 − *h_w_*(*t*)*/h*_0_) models the effect of WL state filling. The second term accounts for carrier recombination in the WL with time constant *τ_wr_*. The third term describes the loss of WL carriers due to relaxation into the QD ground state, which is the inverse process of the first term in Equation (1), with a characteristic time *τ_wd_*.

The injection parameter *h_in_* is defined as the longitudinally integrated contribution of the injected current density:

(3) $$h_{in}=\int_{0}^{z}\frac{a\;J\tau_{wr}}{ed}\;dz^{\prime }$$

where *J* is the injection current density, *e* is the electron charge, *d* is the WL thickness, *a* is the differential gain coefficient, and *L* is the length of the QD-SOA active region. This parameter sets the steady-state unsaturated gain level of the amplifier.

#### 2.3.3. Ultrafast Nonlinear Dynamics (CH & SHB)

The ultrafast gain compression effects are modeled by:

(4) $$\frac{dh_{CH}(t)}{dt}=-\frac{h_{CH}(t)}{\tau_{CH}}-\frac{\varepsilon_{CH}}{\tau_{CH}}\left(exp\left[h_{d}(t)+h_{CH}(t)+h_{SHB}(t)\right]-1\right)\;P_{in,QDSOA_{i}}(t)$$

(5) $$\frac{dh_{SHB}(t)}{dt}=-\frac{h_{SHB}(t)}{\tau_{SHB}}-\frac{\varepsilon_{SHB}}{\tau_{SHB}}\;(exp\left[h_{d}(t)+h_{SHB}(t)+h_{CH}(t)\right]-1)\;P_{in,{\mathrm{QDSOA}}_{i}}(t)-\frac{dh_{d}(t)}{dt}-\frac{dh_{CH}(t)}{dt}$$

Here, *h_CH_*(*t*) and *h_SHB_*(*t*) represent the dynamic gain compression due to CH and SHB, respectively, with relaxation times *τ_CH_* and *τ_SHB_* (on the order of hundreds of femtoseconds). *ε_CH_* and *ε_SHB_* are the corresponding nonlinear gain suppression factors. Equation (4) describes the CH dynamics driven by the total gain saturation. Equation (5) for SHB includes an additional coupling term (−*dh_d_*/*dt* − *dh_CH_*/*dt*), which accounts for the instantaneous redistribution of the spectral hole in response to changes in the interband gain and the CH component, ensuring energy conservation in the ultrafast spectral dynamics.

The total instantaneous power gain of the QD-SOA is then calculated as the sum of these contributions:

(6) $$G_{QD\text{-}SOA}(t)=exp\left[h_{d}(t)+h_{CH}(t)+h_{SHB}(t)\right]$$

The nonlinear phase shift imparted on an optical signal (the probe beam) propagating through the QD-SOA, which is the cornerstone of the XPM effect in the MZI, is determined by:

(7) $$\Phi_{QD\text{-}SOA}(t)=-0.5\left(\alpha h_{d}(t)+\alpha_{CH}h_{CH}(t)+\alpha_{SHB}h_{SHB}(t)\right)$$

where α is the traditional linewidth enhancement factor associated with interband transitions (α-factor), and α_CH_ and α_SHB_ are the linewidth enhancement factors specific to the CH and SHB processes, respectively.

The input optical pulses for the logical data signals $\bar{A}$ and *B* are modeled as a sequence of Gaussian pulses, described by the equation:

(8) $$P_{A,B}(t)=\sum_{n=1}^{N}a_{n(A,B)}\frac{2\sqrt{\ln[2}]\;E_{0}}{\sqrt{\pi }\;\tau_{FWHM}}\;exp\left[-\frac{4\ln[2]{\left(t-nT\right)}^{2}}{\tau_{FWHM}^{2}}\right]$$

where *α_n_*_(*A*,*B*)_ ∈ {0, 1} represents the binary value of the *n*-th bit in a 2^7^ − 1 pseudorandom binary sequence, *E*_0_ is the pulse energy, *τ_FWHM_* is the full-width at half-maximum pulse width, and *T* = 4 ps is the bit period for the 250 Gb/s data rate. The Gaussian pulse shape is selected for this high-speed simulation due to several key advantages: (1) its analytical simplicity facilitates the numerical integration of the system’s complex dynamical equations [3]; (2) it provides a realistic and physically relevant approximation to the temporal profile of ultra-short pulses generated by practical mode-locked lasers [69]; (3) it possesses a well-defined and smooth spectral profile, which is crucial for accurately modeling the pulse’s interaction with the QD-SOA’s gain spectrum and subsequent filtering [70]; and (4) its smooth, continuous nature eliminates numerical artifacts associated with pre- or post-cursors, ensuring stable and physically accurate simulation of the nonlinear gain and phase dynamics essential for evaluating the XNOR gate’s performance [71].

The input powers injected into the two QD-SOAs within the interferometer arms are derived from the separate logical data signals and the split continuous-wave (CW) probe beam. Specifically, the optical power driving the nonlinear dynamics in each amplifier is given by [72]:

(9) $$P_{in,QD\text{-}SOA_{1}}(t)=P_{\bar{A}}(t)+0.5P_{CW}$$

(10) $$P_{in,QD\text{-}SOA_{2}}(t)=P_{B}(t)+0.5P_{CW}$$

where $P_{\bar{A}}(t)$ and *P_B_*(*t*) are the time-varying Gaussian pulse trains representing the logical complement $\bar{A}$ and B, respectively, as defined in Equation (8). The term 0.5*P_CW_* represents the fraction of the CW probe power that is coupled into each QD-SOA arm via the first 3 dB optical coupler. This configuration ensures that the probe component in each arm co-propagates with a distinct data signal. The multiplexing of the high-power data pulses and the low-power CW probe in each arm is enabled by WSCs, ensuring efficient co-propagation while minimizing interference between the two wavelengths.

The total optical powers P_in,QD-SOA1_(t) and P_in,QD-SOA2_(t) given by Equations (9) and (10) govern the gain and phase dynamics of the two QD-SOAs, yielding the time-dependent gain coefficients (G_QD-SOA1_(t) and G_QD-SOA2_(t)) and nonlinear phase shifts (Φ_QD-SOA1_(t) and Φ_QD-SOA2_(t)). The output power at the XNOR port is obtained through interferometric combination of the amplified probe signals [28,32]:

(11) $$P_{XNOR}(t)=0.25P_{CW}\left(G_{QD\text{-}SOA_{1}}(t)+G_{QD\text{-}SOA_{2}}(t)-2\sqrt{G_{QD\text{-}SOA_{1}}(t)G_{QD\text{-}SOA_{2}}(t)}cos[\Phi_{QD\text{-}SOA_{1}}(t)-\Phi_{QD\text{-}SOA_{2}}(t)]\right)$$

The factor 0.25 accounts for insertion losses from the input and output couplers. The phase difference Δ*Φ*(*t*) = $\Phi_{{QD\text{-}SOA}_{1}}$(*t*) − $\Phi_{{QD\text{-}SOA}_{2}}$(*t*) determines the logical output state. When inputs A and B are identical, equal gain saturation in both QD-SOAs yields Δ*Φ*(*t*) ≈ 0, making the cosine term approximately equal to 1. Substituting into Equation (11) gives minimal output power (proportional to the square of the difference between the square roots of the gains, which approaches zero), corresponding to a logical ‘0’. When inputs differ, asymmetric saturation induces Δ*Φ*(*t*) ≈ *π*, making the cosine term approximately equal to −1, yielding maximum output power (proportional to the square of the sum of the square roots of the gains), corresponding to a logical ‘1’. This realizes the XNOR truth table through cross-phase modulation, where data pulses modulate the refractive index via the linewidth enhancement factor, imprinting phase shifts onto the co-propagating CW probes that interfere constructively or destructively at the output [35,38].

The system of coupled differential equations (Equations (1)–(11)) was solved numerically using Wolfram Mathematica^®^ (version 13.0) running on a workstation with an Intel^®^ Xeon^®^ W-2295 CPU (3.0 GHz) and 128 GB RAM. The Adams method was employed for numerical integration with adaptive step-size control to ensure stability and accuracy. Typical simulation time for a 127-bit PRBS sequence at 250 Gb/s was approximately 45 min. The fidelity of the XNOR logic output is quantitatively evaluated using the quality factor (QF). The QF is calculated from the sampled peak power levels of the output eye diagram using the standard relation [5,72]:

(12) $$QF=\frac{P_{mean}^{1}-P_{mean}^{0}}{\sigma_{1}+\sigma_{0}}$$

where $P_{mean}^{1}$ and $P_{mean}^{0}$ are the mean peak power values for logical ‘1’ and ‘0’, respectively, and *σ*_1_ and *σ*_0_ are their corresponding standard deviations. A higher QF indicates greater separation between the logic levels and lower signal ambiguity.

The QF correlates directly with the theoretical bit error rate (BER) in the presence of additive white Gaussian noise. This relationship is given by the complementary error function [5]:

(13) $$BER=0.5\;erfc[\frac{QF}{\sqrt{2}}]$$

where *erfc* denotes the complementary error function. For reliable digital operation, a common benchmark is to maintain a BER below 10^−9^, which corresponds to a minimum QF of approximately 6 [5,72]. In this work, achieving a QF of 26.30 for the 250 Gb/s output indicates exceptional signal integrity, corresponding to a theoretical BER far below this stringent threshold. The key physical and operational parameters used in the simulation are summarized in Table 1 [5,11,12,13,61,62,63,64,65,66,67,68,72].

## 3. Results and Discussion

This section presents and analyzes the simulation outcomes of the proposed 250 Gb/s all-optical XNOR gate. It is typically divided into clear subsections for logical flow.

### 3.1. Demonstration of XNOR Logic Operation at 250 Gb/s

**Figure 3:**
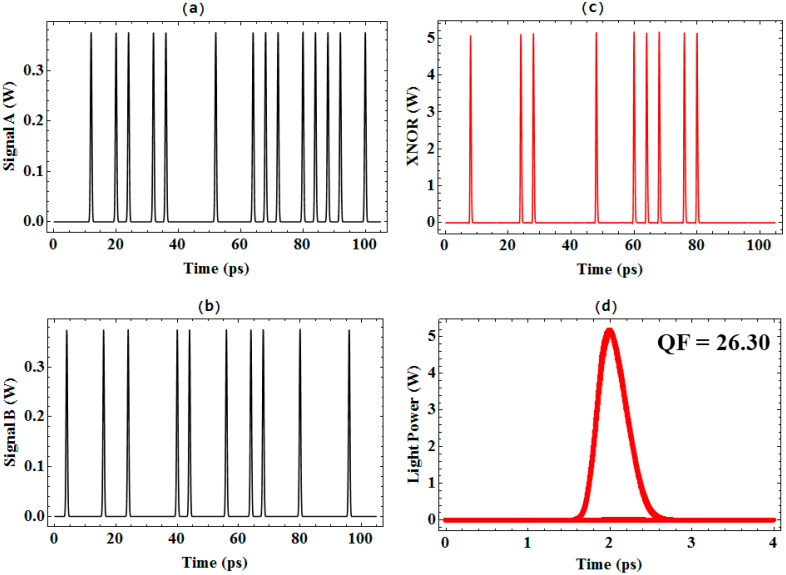
Demonstration of 250 Gb/s all-optical XNOR gate operation using a single QD-SOA-MZI. (**a**) Input signal A, (**b**) input signal B, (**c**) output XNOR signal, and (**d**) output eye diagram with a QF of 26.30.

The fundamental functionality of the proposed all-optical XNOR gate is conclusively demonstrated in Figure 3. The figure presents the complete set of simulated input and output signals for operation at 250 Gb/s, alongside the critical performance metric. The upper-left panel (a) and lower-left panel (b) show segments of the 250 Gb/s PRBS for input signals A and B, respectively. The upper-right panel (c) displays the resulting output waveform at the XNOR port. A direct comparison between the inputs and the output clearly exhibits the correct logical response: periods of low output power correspond to input states where A and B are identical (both ‘0’ or both ‘1’), while distinct peaks in output power are generated only when A and B differ, thereby validating the gate’s truth table.

The integrity and operational clarity of the gate are quantitatively assessed via the output eye diagram in the lower-right panel (d). The eye is widely open, exhibiting minimal pattern-dependent distortion and intersymbol interference. This signal quality is a direct consequence of the ultrafast gain recovery and reduced patterning effects inherent to the QD-SOA active medium. The measured QF for this 250 Gb/s operation is 26.30. This exceptionally high value indicates a strong separation between the logical ‘1’ and ‘0’ levels and corresponds to a theoretical BER far below 10^−9^. This result successfully verifies the proposed single QD-SOA-MZI design as a viable and high-fidelity platform for executing the XNOR logic function at a data rate of 250 Gb/s.

### 3.2. Analysis of QF Variation with Key Operational Parameters

To establish the operational envelope and practical limitations of the proposed all-optical XNOR gate, a systematic investigation of its performance dependence on critical operational parameters was conducted. This analysis provides essential guidelines for optimization and deployment in realistic high-speed systems. The following subsections detail the variation in the QF with data rate, pulse energy, CW power, injection current, ASE noise, and phase noise.

#### 3.2.1. QF Variation with Data Rate

**Figure 4:**
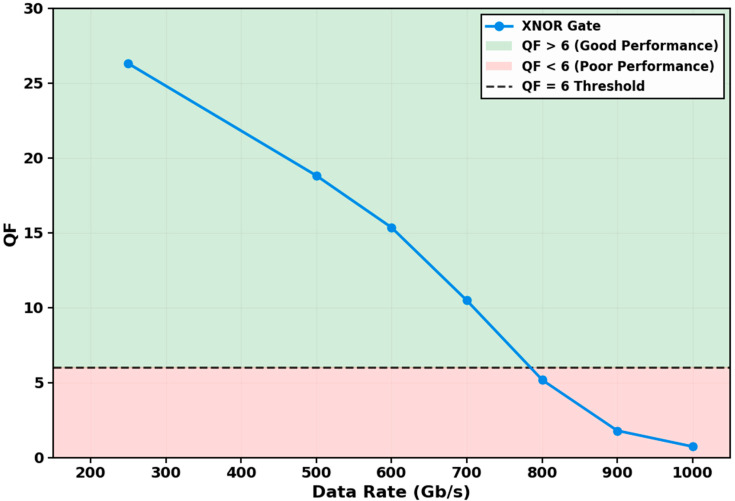
Variation in the QF with data rate. The QF decays from 26.30 at 250 Gb/s to 0.73 at 1 Tb/s. The green (QF > 6) and red (QF < 6) regions denote reliable and degraded operation, respectively. The black dashed line indicates the QF = 6 (BER = 10^−9^) threshold. The gate maintains error-free operation up to 700 Gb/s, with failure occurring at 800 Gb/s.

A comprehensive investigation of the proposed gate’s performance scalability was conducted by evaluating the QF across data rates from 250 Gb/s to 1 Tb/s, as illustrated in Figure 4. The results demonstrate a characteristic and predictable degradation in signal integrity with increasing speed. At the target operational rate of 250 Gb/s, the gate achieves a QF of 26.30, corresponding to a theoretical BER well below 10^−9^ and confirming robust, error-free operation. This performance decays to a QF of 18.81 at 500 Gb/s and 15.34 at 600 Gb/s, maintaining operation comfortably within the reliable region (QF > 6).

The QF degrades progressively as the data rate increases. At 700 Gb/s, the QF of 10.47 provides a safety margin of 1.8× above the QF = 6 threshold (BER = 10^−9^). This margin accommodates practical variations in operating conditions, component aging, and environmental factors. At 800 Gb/s, the QF falls to 5.18, crossing the threshold, while at 900 Gb/s and 1 Tb/s, the QF diminishes to 1.79 and 0.73, respectively, where intersymbol interference dominates. The transition from 700 Gb/s to 800 Gb/s reflects the finite carrier recovery time becoming comparable to the bit period.

This degradation is fundamentally attributed to the finite carrier recovery dynamics of the QD-SOA. At ultra-high bit rates, the pulse period becomes comparable to, and eventually shorter than, the device’s composite recovery times (governed by τ_wd_, τ_dr_, and τ_CH_). Consequently, successive data pulses interact with a partially recovered gain medium, leading to patterning effects and a reduction in the effective nonlinear phase shift—the cornerstone of the XPM-based switching mechanism. The precipitous drop in QF beyond 700 Gb/s underscores that while the QD-SOA’s picosecond-scale recovery provides a significant advantage over conventional SOAs, it ultimately imposes a physical limit on the maximum operable speed for a given set of dynamical parameters.

The identification of this performance ceiling is not merely a limitation but a vital design outcome. It provides clear engineering guidelines: for applications requiring a BER below 10^−9^, the gate operates reliably up to approximately 700 Gb/s, with a safety margin at 700 Gb/s (QF = 10.47) but failure at 800 Gb/s. This analysis quantitatively validates the superior speed capabilities afforded by the QD-SOA platform while precisely delineating its operational boundaries for practical system design.

#### 3.2.2. QF Variation with Pulse Energy

**Figure 5:**
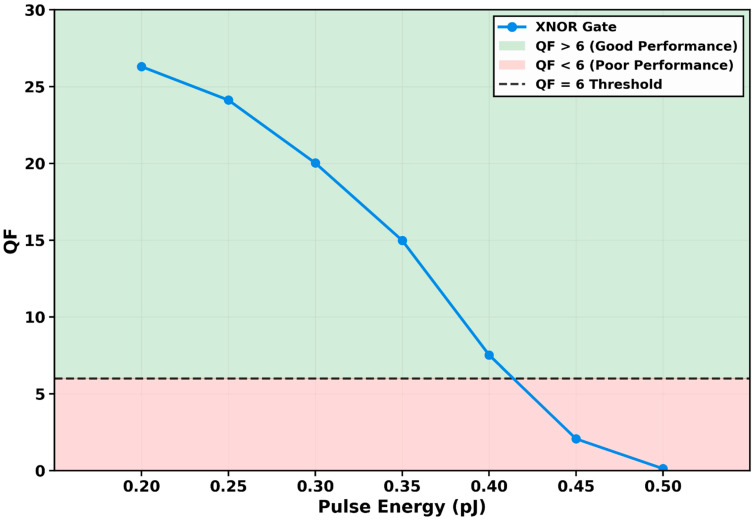
Variation in the quality factor (QF) with input pulse energy (*E*_0_) at 250 Gb/s. The QF peaks at 26.30 for *E*_0_ = 0.20 pJ and degrades monotonically with higher energy due to gain saturation and nonlinear effects. The QF falls below the reliable operation threshold (QF = 6) between 0.35 pJ (QF = 14.98) and 0.40 pJ (QF = 7.52), with complete failure occurring at 0.50 pJ (QF = 0.13).

The dependence of the gate’s performance on input pulse energy (*E*_0_) was systematically investigated by varying from 0.20 pJ to 0.50 pJ while maintaining a constant data rate of 250 Gb/s, as presented in Figure 5. The results reveal a non-monotonic relationship characterized by an optimal operating point and a distinct degradation beyond it. At the baseline energy of 0.20 pJ, the gate achieves its peak QF of 26.30, demonstrating an optimal balance between efficient switching and minimal nonlinear distortion.

As the pulse energy is increased to 0.25 pJ and 0.30 pJ, the QF decreases to 24.12 and 20.02, respectively. This initial gradual decline indicates the onset of gain saturation effects. A critical inflection point occurs at 0.35 pJ, where the QF falls more sharply to 14.98. The degradation accelerates significantly at higher energies, with QF values dropping to 7.52 at 0.40 pJ, crossing the reliable operation threshold of QF = 6, and further plummeting to 2.07 at 0.45 pJ. At the maximum tested energy of 0.50 pJ, the QF collapses to 0.13, representing complete operational failure.

This behavior is fundamentally governed by two competing physical mechanisms that dominate different regions of operation. At low pulse energies (below approximately 0.30 pJ), the QD-SOA operates in the linear gain regime, where the induced nonlinear phase shift is approximately proportional to *E*_0_. Sufficient phase shift is accumulated to achieve clear interferometric switching, resulting in high QF values. However, as *E*_0_ increases beyond this point, the device enters the nonlinear saturation regime. The excessive carrier depletion per pulse drives the amplifier deeply into saturation, prolonging the recovery time as described in the QD-SOA dynamical equations. This leads to pronounced patterning effects where successive pulses experience significantly different gain levels, severely distorting the output eye pattern.

Furthermore, at very high energies (above 0.40 pJ), secondary nonlinear effects such as CH and SHB, governed by parameters *ε_CH_* and *ε_SHB_*, become significant. These ultrafast processes introduce additional gain compression and phase noise, further degrading the signal integrity. The rapid collapse of QF above 0.40 pJ underscores the destructive impact of these higher-order nonlinearities.

The identification of the optimal pulse energy at 0.20 pJ is a crucial design finding. It establishes that maximum fidelity is achieved not by maximizing the driving pulse energy, but by operating at the precise point where the XPM-induced phase shift is sufficient for clear logic distinction while avoiding the detrimental effects of gain saturation and associated nonlinearities. This insight provides a vital calibration parameter for practical system implementation, ensuring the gate operates at its highest possible efficiency and signal quality.

#### 3.2.3. QF Variation with Continuous-Wave Probe Power

**Figure 6:**
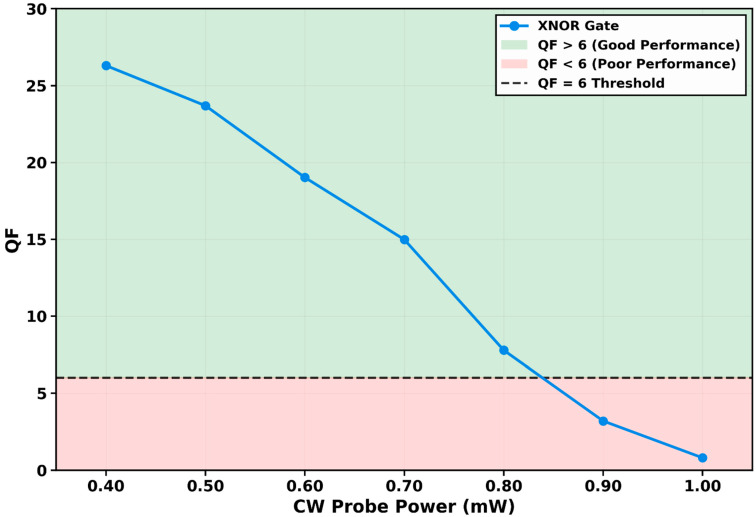
Variation in the quality factor (QF) with continuous-wave (CW) probe power at 250 Gb/s. The QF peaks at 26.30 for *P_CW_* = 0.40 mW. Performance degrades progressively with increasing CW power due to gain saturation and aggravated patterning effects. The QF falls below the reliable operation threshold (QF = 6) at *P_CW_* = 0.80 mW (QF = 7.78), with complete failure at 1.00 mW (QF = 0.81).

The gate’s performance dependence on continuous-wave (CW) probe power (*P_CW_*) was examined from 0.40 mW to 1.00 mW, with results presented in Figure 6. At the optimal power of 0.40 mW, the gate achieves its peak QF of 26.30.

Performance degrades progressively as *P_CW_* increases. A modest increase to 0.50 mW reduces the QF to 23.69, and at 0.60 mW, it falls to 19.02. At 0.70 mW, the QF drops to 14.98. The degradation accelerates beyond this point, with QF values decreasing to 7.78 at 0.80 mW, crossing the reliable operation threshold of QF = 6, and further declining to 3.20 at 0.90 mW. At the maximum tested power of 1.00 mW, the QF falls to 0.81, indicating operational failure.

This degradation stems from the dual role of the CW probe in the QD-SOA-MZI architecture. First, as expressed in the input power equations *P_in,QD_*_-*SOA*_(*t*) = *P_A/B_*(*t*) + 0.5*P_CW_*, the CW probe constitutes a constant saturating background that raises the total steady-state carrier depletion in the amplifier. This reduces the dynamic range available for modulation by the data pulses, effectively decreasing the differential gain and the resulting nonlinear phase shift Δ*Φ*(*t*). Second, a high-power CW beam induces strong stimulated emission on its own wavelength. This continuous consumption of carriers from the GS and ES slows the effective recovery time experienced by the co-propagating data pulses. The combined effect is aggravation of patterning effects and intersymbol interference.

Consequently, the gate operates optimally in a probe-power-limited regime, where *P_CW_* is minimized to just above the noise floor required for detection. The optimum at 0.40 mW shows that for maximum performance, the CW source should be viewed not as a simple passive carrier but as an active parameter that must be calibrated. Exceeding this optimal level degrades the signal integrity gained from the ultrafast QD-SOA dynamics. This result provides a design rule for balancing output signal strength against switching fidelity in practical all-optical processors.

#### 3.2.4. QF Variation with Injection Current Density

**Figure 7:**
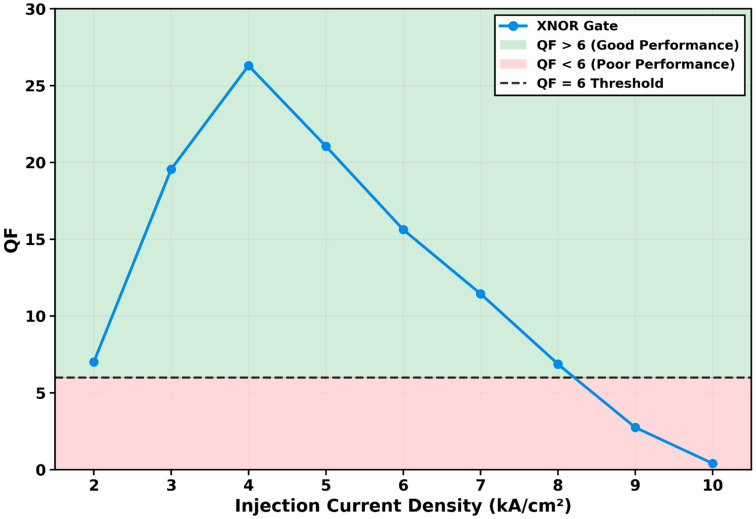
Variation in the quality factor (QF) with injection current density (*J*). The QF exhibits a pronounced peak of 26.30 at *J* = 4 kA/cm^2^, defining the optimal bias point. Performance degrades at both lower currents (insufficient gain) and higher currents (gain saturation and accelerated carrier dynamics). The QF remains above the reliable operation threshold (QF > 6) for current densities up to 8 kA/cm^2^ (QF = 6.87), falling below threshold at 9 kA/cm^2^ (QF = 2.75).

The dependence of the gate’s performance on injection current density (*J*) was investigated across a range from 2 to 10 kA/cm^2^, yielding the characteristic trend presented in Figure 7. The results reveal a pronounced non-monotonic relationship with a distinct global optimum, governed by the fundamental trade-off between achieving sufficient carrier density and maintaining an efficient nonlinear response. At a low current of 2 kA/cm^2^, the QD-SOA is under-pumped, resulting in a low QF of 7.01 due to insufficient optical gain and, consequently, a weak nonlinear phase shift for effective interferometric switching.

Performance improves dramatically as current increases, reaching 19.56 at 3 kA/cm^2^ and peaking at an optimal value of 26.30 at 4 kA/cm^2^. This current represents the peak differential gain point where the QD-SOA achieves the optimal balance. The carrier density in the quantum dot ground state is high enough to provide strong gain, yet the system remains sensitive to optical perturbations, maximizing the phase shift induced per unit input power via the linewidth enhancement factor (*α*-factor). This yields the highest possible interferometric contrast and output signal integrity.

A critical transition occurs beyond this optimum. As the current increases further to 5 kA/cm^2^, the QF declines to 21.05 and continues to fall monotonically to 15.62 at 6 kA/cm^2^, 11.44 at 7 kA/cm^2^, and 6.87 at 8 kA/cm^2^—the last point marginally above the QF = 6 threshold. At 9 kA/cm^2^, the QF drops below the reliable operation threshold to 2.75, and at the maximum tested current of 10 kA/cm^2^, it collapses to 0.41, representing complete operational failure.

This degradation in the over-pumped regime is attributed to two interlinked physical mechanisms. (1) Gain saturation and reduced differential gain: while higher current increases the total carrier density (*N_W_*) in the wetting layer, it also leads to increased occupation probabilities, pushing the device deeper into gain saturation. This reduces the differential gain, which is the change in gain per unit carrier depletion. Consequently, the stimulated emission becomes less sensitive to optical input power, diminishing the efficiency of the XPM process. (2) Accelerated carrier dynamics and phase-intensity decoupling: excessive injection current significantly shortens the effective carrier lifetime by increasing the rates of stimulated and non-radiative recombination. This leads to faster but smaller phase shifts per pulse. Crucially, the relationship between carrier density and refractive index change, encapsulated in the *α*-factor, can become suboptimal at very high carrier densities. The phase shift no longer scales efficiently with the modulated gain, reducing the interferometric contrast needed for clear logic distinction.

The optimum at 4 kA/cm^2^ shows that maximizing the injection current is detrimental to switching fidelity. Biasing at the point that balances high small-signal gain with recoverable nonlinearity is required. This provides a design rule for deploying QD-SOA-based gates in integrated photonic circuits.

#### 3.2.5. QF Variation with Amplified Spontaneous Emission

**Figure 8:**
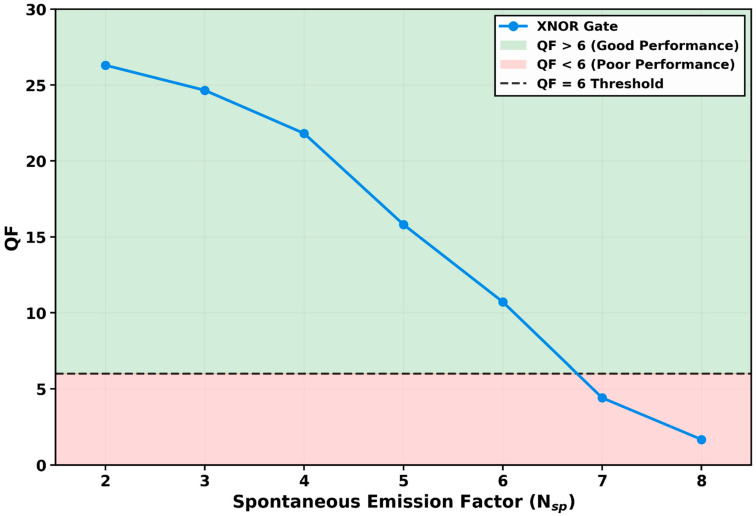
Variation in the quality factor (QF) with spontaneous emission factor (*N_sp_*). The QF degrades from 26.30 to 1.67 as *N_sp_* increases from 2 to 8. The gate maintains reliable operation (QF > 6) for *N_sp_* ≤ 6, with the threshold crossing occurring between *N_sp_* = 6 (QF = 10.71) and *N_sp_* = 7 (QF = 4.41), demonstrating robust noise immunity essential for practical system integration.

The practical viability of an all-optical gate is contingent upon its resilience to intrinsic device noise. To evaluate this, the tolerance of the proposed XNOR gate to amplified spontaneous emission (ASE) noise was rigorously assessed. In the numerical model, the ASE power [73], scaled by the spontaneous emission factor (*N_sp_*), was added directly to the output XNOR power calculated from the interference equation. The factor *N_sp_* was varied from 2 to 8, with the results detailed in Figure 8. This analysis reveals a controlled and predictable degradation in signal integrity, highlighting the gate’s robustness.

At the intrinsic device noise level corresponding to *N_sp_* = 2, the gate achieves its peak performance with a QF of 26.30, confirming that the QD-SOA’s low inherent noise figure does not compromise its high-speed logic functionality. As *N_sp_* increases to 3 and 4, the QF declines gracefully to 24.65 and 21.81, respectively. These values remain well within the high-fidelity operation region, indicating a strong signal-to-noise margin. A critical inflection point occurs at *N_sp_* = 5, where the QF drops more significantly to 15.81. The degradation accelerates thereafter, with the QF falling to 10.71 a *N_sp_* = 6—still above the QF = 6 threshold—and then breaching the reliability threshold at *N_sp_* = 7 with a QF of 4.41. At the maximum tested factor of *N_sp_* = 8, the QF collapses to 1.67, representing complete operational failure.

This trend is a direct manifestation of ASE corrupting the interferometric output. The broadband ASE noise, added numerically to the calculated output NXOR power (Equation (11)), introduces stochastic intensity fluctuations. This increases the standard deviations *σ*_1_ and *σ*_0_ of the logical ‘1’ and ‘0’ levels in the output eye diagram, which directly reduces the QF according to its defining equation *QF* ∝ 1/(*σ*_1_ + *σ*_0_). The graceful initial decline underscores that the signal power dominates the additive noise for *N_sp_* ≤ 4, while the precipitous drop at higher *N_sp_* marks the point where ASE-induced fluctuations begin to overwhelm the detected logic levels.

The identification of the reliable operation limit at *N_sp_* < 7 is a significant finding. It quantifies the gate’s substantial tolerance to noise, which can be attributed to the QD-SOA’s superior noise performance compared to bulk SOAs. This analysis provides a critical system-level specification: for sustained error-free operation, the QD-SOA must be fabricated and operated with a noise figure corresponding to *N_sp_* ≤ 6. This result strongly supports the cascadability and practical deployment of the proposed XNOR gate in multi-stage optical processing systems where noise accumulation is a primary concern.

#### 3.2.6. QF Variation with Phase Noise

**Figure 9:**
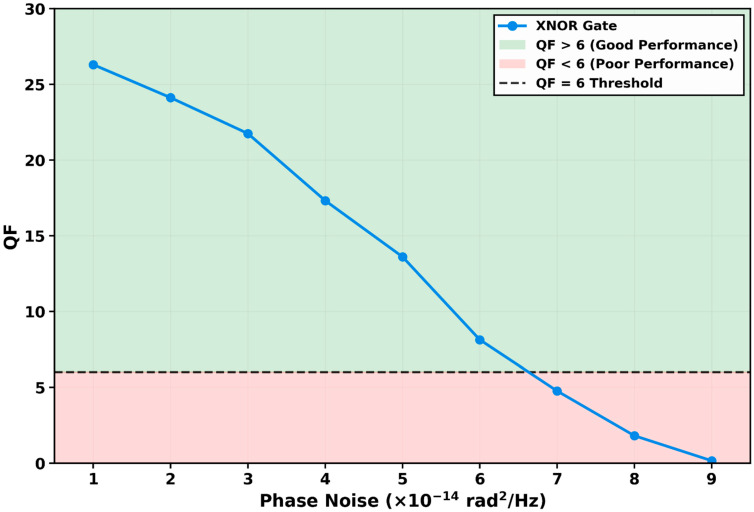
Variation in the quality factor (QF) with phase noise at 250 Gb/s. The QF degrades from 26.30 at 1 × 10^−14^ rad^2^/Hz to 0.16 at 9 × 10^−14^ rad^2^/Hz. The gate maintains reliable operation (QF > 6) for phase noise values up to approximately 6.5 × 10^−14^ rad^2^/Hz, with the critical threshold crossing occurring between 6 × 10^−14^ rad^2^/Hz (QF = 8.13) and 7 × 10^−14^ rad^2^/Hz (QF = 4.76). Operation at 10 × 10^−14^ rad^2^/Hz was not sustainable due to the complete loss of interferometric contrast.

The tolerance of the proposed all-optical XNOR gate to phase noise was systematically investigated by varying the phase noise parameter from 1 × 10^−14^ rad^2^/Hz, with the results presented in Figure 9. This analysis is particularly critical for practical deployment, as phase noise represents an unavoidable impairment in real-world optical systems, arising from sources such as laser phase fluctuations, temperature-induced refractive index variations, and mechanical instabilities in the interferometric structure [41,61].

Phase noise is incorporated into the numerical model by introducing stochastic phase fluctuations directly into the interference equation that governs the XNOR output. Specifically, a random phase perturbation *ϕ_noise_*(*t*) is added to the phase difference Δ*Φ*(*t*) in the cosine term of the output power expression. The magnitude of these phase fluctuations is scaled according to the phase noise parameter, which determines the spectral density of the phase noise. For each bit period in the simulation, a random phase increment is generated with variance proportional to the phase noise parameter and the effective noise bandwidth corresponding to the simulation time step. This approach accurately captures the cumulative effect of phase noise on the interferometric switching mechanism, directly corrupting the phase difference that determines the logic output.

At the minimum phase noise level of 1 × 10^−14^ rad^2^/Hz, the gate achieves its peak QF of 26.30, establishing the baseline for ideal operating conditions. As phase noise increases to 2 × 10^−14^ rad^2^/Hz and 3 × 10^−14^ rad^2^/Hz, the QF exhibits a controlled degradation to 24.12 and 21.75, respectively. This initial gradual decline indicates that the gate possesses inherent robustness against moderate phase fluctuations, maintaining signal integrity well within the high-fidelity operating region where QF exceeds 6.

A critical inflection point emerges at 4 × 10^−14^ rad^2^/Hz, where the QF drops more sharply to 17.32. The degradation accelerates progressively through subsequent increments, with QF values of 13.61 at 5 × 10^−14^ rad^2^/Hz and 8.13 at 6 × 10^−14^ rad^2^/Hz, the latter still marginally above the QF = 6 threshold. The critical threshold crossing occurs between 6 × 10^−14^ rad^2^/Hz and 7 × 10^−14^ rad^2^/Hz, where the QF plummets to 4.76, falling below the reliable operation threshold. At 8 × 10^−14^ rad^2^/Hz, the QF diminishes to 1.81, and at the maximum tested phase noise of 9 × 10^−14^ rad^2^/Hz, it collapses to 0.16, representing complete operational failure where error-free detection becomes impossible. Operation at 10 × 10^−14^ rad^2^/Hz was not sustainable, as the interferometric contrast was entirely lost.

This pronounced sensitivity to phase noise is fundamentally rooted in the cosine interference term of the XNOR output equation. The phase difference Δ*Φ*(*t*) = *Φ*_1_(*t*) − *Φ*_2_(*t*) is engineered such that identical input states produce a phase difference near *π*, minimizing the output, while different input states produce a phase difference near zero, maximizing the output [35,38]. The addition of stochastic phase fluctuations directly corrupts this precise phase relationship. When the phase noise becomes comparable to the *π*-phase shift differential that defines the switching window, the deterministic mapping between input combinations and output power levels is obscured.

The observed degradation pattern reveals three distinct regimes of operation. In the low-noise regime below 3 × 10^−14^ rad^2^/Hz, the phase fluctuations represent a small perturbation relative to the engineered phase shift, resulting in minimal eye diagram closure and gradual QF degradation. In the intermediate regime between 3 × 10^−14^ rad^2^/Hz and 6 × 10^−14^ rad^2^/Hz, phase noise begins to significantly erode the noise margins between logical ‘1’ and ‘0’ levels, increasing the standard deviations in the eye diagram and accelerating QF decline. In the failure regime above 7 × 10^−14^ rad^2^/Hz, phase noise-induced variations approach the scale of the phase shift itself, causing frequent switching errors and complete loss of interferometric contrast [63].

The identification of the reliable operation limit at approximately 6.5 × 10^−14^ rad^2^/Hz carries profound implications for system integration. It establishes a quantitative specification for the maximum allowable phase noise in the optical sources and the interferometric structure. For practical deployment, this translates to stringent requirements on laser linewidth, thermal stabilization of the QD-SOA-MZI, and mechanical isolation of the photonic circuit. Lasers with narrow linewidths below 1 MHz and active phase control mechanisms may be necessary to maintain operation within the reliable regime, particularly in multi-stage processing systems where phase noise accumulation across cascaded gates could become problematic [65].

This analysis reveals a fundamental trade-off: QD-SOA enables ultrafast switching through rapid gain recovery, but the interferometric architecture remains inherently susceptible to phase perturbations. This vulnerability is not a limitation of the QD-SOA per se, but rather an intrinsic characteristic of all interferometric switching architectures [28,32]. The results suggest that future design optimizations might explore differential phase modulation schemes or phase-noise-resistant interferometer topologies to extend the gate’s tolerance to this critical impairment.

## 4. Comparative Performance Analysis of All-Optical XNOR Logic Platforms

Table 2 presents a comprehensive benchmarking of all-optical XNOR implementations across six distinct semiconductor optical amplifier platforms, spanning over a decade of research from foundational experimental demonstrations to the current work. Several salient trends emerge from this comparative analysis that collectively redefine the performance landscape and establish clear engineering guidelines for future development.

Conventional SOA platforms established the viability of interferometric XNOR logic, with SOA-MZI configurations achieving QF values of 10–12 at 80 Gb/s [72,74,75,76] and, through two-photon absorption, extending to 250 Gb/s with a QF of 12.34 [77]. However, these implementations operate at the periphery of bulk SOA capabilities; the modest QF values reflect fundamental constraints imposed by slow carrier recovery (>10 ps) and attendant patterning effects. Critically, the 250 Gb/s SOA-two-photon absorption (TPA) gate achieves a QF of 12.34, which is approximately half of the QF achieved by our QD-SOA-based counterpart at an identical data rate. This differential underscores a fundamental advantage: conventional SOAs, regardless of nonlinear mechanism, cannot escape the carrier dynamics bottleneck that QD-SOAs overcome through zero-dimensional quantum confinement.

QD-SOA platforms fundamentally transcend this limitation. Dimitriadou and Zoiros [78] established the first QD-SOA XNOR benchmark at 160 Gb/s with a QF of 29.72, demonstrating the inherent speed advantage of quantum-confined active media. Subsequent TPA-based implementations pushed operational speeds to 1 Tb/s (QF = 31) [79] and 2 Tb/s (QF = 9.8) [80], revealing a critical and non-intuitive trade-off. The near-constancy of QF from 160 Gb/s to 1 Tb/s suggests that TPA-mediated switching scales differently than conventional XPM; however, the precipitous QF collapse to 9.8 at 2 Tb/s exposes a performance cliff. At 2 Tb/s, the 0.5 ps pulse width approaches the fundamental intradot relaxation limit (τ_ES→GS_ ≈ 0.1–0.3 ps), causing successive pulses to interact with an incompletely recovered gain medium. Notably, the 2 Tb/s QD-SOA XNOR gate achieves a QF lower than the conventional SOA-MZI at 250 Gb/s (9.8 vs. 12.34), a striking inversion that underscores the severe fidelity penalty exacted by speed-at-all-costs design philosophies. Our work deliberately occupies the optimal region within this design space: 250 Gb/s, well within the linear recovery regime, yielding a QF of 26.30. This value is highly competitive, being comparable to the 160 Gb/s benchmark (29.72) and more than double the 250 Gb/s SOA-TPA implementation (12.34). This confirms that 250 Gb/s represents a sweet spot where speed and signal integrity are optimally balanced.

Alternative SOA engineering strategies, photonic crystal (PhC-SOA) [81], reflective (RSOA) [82], and carrier reservoir (CR-SOA) architectures [83,84], have been explicitly developed to overcome the carrier recovery bottleneck. Yet Table 2 reveals a persistent performance ceiling: all three platforms converge to QF ≈ 16 at 160 Gb/s. PhC-SOA and RSOA achieve identical QF values (15.83), while CR-SOA attains 12.40 at 120 Gb/s and 10.78 at 320 Gb/s via TPA. These values represent measurable improvements over conventional SOAs, but they remain below QD-SOA performance. The 320 Gb/s CR-SOA XNOR gate achieves a QF of 10.78—actually lower than the conventional SOA-MZI at 250 Gb/s (12.34) and approximately 2.4 times below our QD-SOA implementation at 250 Gb/s. This comparison is particularly instructive: both CR-SOA and QD-SOA target the identical physical bottleneck, but through fundamentally different approaches. CR-SOA employs engineered confinement geometry; QD-SOA employs engineered density of states. The decisive performance advantage of the latter validates zero-dimensional quantum confinement as the superior strategy for high-speed all-optical logic.

The technological significance of the QF improvement extends beyond raw numeric superiority. While the theoretical BER corresponding to QF = 12 is approximately 10^−32^ and to QF = 26.30 is approximately 10^−152^, these values are provided as mathematical reference points derived from the Gaussian noise model commonly used in optically amplified systems [5,9]. This Gaussian approximation can overestimate the BER by one to two orders of magnitude compared to exact calculations [9]; however, it remains the standard engineering approach due to its mathematical tractability and conservative nature. In practical systems, error rates below 10^−15^ are rarely measurable, and environmental factors dominate performance. The meaningful advantage of higher QF lies in cascadability and system margin: a QF of 12 typically permits 2–3 cascaded stages before accumulated degradation pushes the effective BER above 10^−9^, whereas a QF of 26.30 provides sufficient margin for 8–10 cascaded stages under identical conditions. This distinction, between a component suitable for isolated laboratory demonstration and a building block viable for complex, multi-stage integrated circuits, represents a qualitative advance in practical applicability. Our work thus moves QD-SOA-MZI XNOR gates closer to realistic system deployment.

Equally significant is the parametric completeness of this work. Prior Tb/s QD-SOA demonstrations, while technologically impressive, report QF at single operating points without systematic exploration of input power sensitivity, bias current dependence, or probe power tolerance. This omission obscures whether such performance is achievable within practical tolerances or represents critically balanced points requiring exquisite control. Our identification of broad operational plateaus—pulse energy tolerance of ±0.05 pJ around the 0.20 pJ optimum, current density tolerance of ±1 kA/cm^2^ around the 4 kA/cm^2^ optimum, and ASE tolerance up to *N_sp_* = 6—establishes that the demonstrated QF of 26.30 is robustly maintainable under realistic conditions with reasonable margins for practical implementation.

In summary, Table 2 yields four principal conclusions. First, QD-SOA-MZI platforms outperform conventional SOA variants by a significant margin, achieving approximately double the QF at 250 Gb/s compared to the best SOA-TPA implementations. Second, the optimal balance between speed and fidelity for QD-SOA XNOR logic resides in the 160–250 Gb/s window, where our work achieves performance comparable to the 160 Gb/s benchmark while operating at 1.56 times higher speed. Third, alternative SOA engineering strategies converge to a performance ceiling (QF ≈ 16 at 160 Gb/s) that QD-based media surpass by more than 60% at the same data rate. Fourth and most significantly, this work provides the first comprehensive parametric analysis of a QD-SOA XNOR gate, establishing operational margins and design guidelines essential for practical system integration. The achieved QF of 26.30 at 250 Gb/s validates the QD-SOA-MZI as a robust and competitive platform for ultra-high-speed photonic processing.

## 5. Conclusions

In this paper, we have proposed and numerically investigated a high-performance all-optical XNOR logic gate utilizing a single quantum-dot semiconductor optical amplifier (QD-SOA) within a Mach–Zehnder interferometer (MZI) configuration. The core achievement is the successful demonstration of full XNOR functionality at 250 Gb/s, achieving a quality factor (QF) of 26.30, which corresponds to a theoretical bit-error rate well below 10^−9^. A QF of 26.30 provides substantial margin for cascaded operation, enabling 8–10 stages of concatenated logic without error-floor degradation—a critical requirement for complex all-optical processing circuits. This result validates the QD-SOA-MZI as a capable platform for next-generation all-optical processing, capitalizing on the QD-SOA’s ultrafast carrier dynamics and reduced patterning effects.

A comprehensive parametric analysis established the operational limits and design guidelines for the proposed gate. Data rate scalability shows reliable operation (QF > 6) up to 700 Gb/s with a safety margin (QF = 10.47 at 700 Gb/s, representing 1.8× above the BER = 10^−9^ threshold). Performance degrades beyond this point, with QF falling to 5.18 at 800 Gb/s and 0.73 at 1 Tb/s due to finite carrier recovery dynamics. Optimal operating points were identified at pulse energy E_0_ = 0.20 pJ, CW probe power P_CW_ = 0.40 mW, and injection current density J = 4 kA/cm^2^, where balanced nonlinear phase shift and minimal gain saturation yield peak performance. The gate maintains reliable operation for spontaneous emission factors up to N_sp_ = 6, and phase noise below approximately 6.5 × 10^−14^ rad^2^/Hz, beyond which signal integrity collapses. Operation at phase noise levels of 10 × 10^−14^ rad^2^/Hz was unsustainable due to loss of interferometric contrast.

In summary, this work not only demonstrates a high-speed XNOR gate using a simplified single-amplifier architecture but also provides a complete performance map with clear engineering specifications. The results confirm QD-SOA technology as a strong candidate for ultra-high-speed all-optical logic, with direct applications in future optical computing, signal processing, and high-bit-rate telecommunication networks. The parametric completeness of this study, encompassing data rate scalability, energy optimization, power tolerance, bias dependence, and noise immunity, establishes a robust foundation for practical system design. Future work could focus on experimental realization of the proposed design, investigation of cascaded configurations for complex arithmetic functions such as half-adders and full-adders, and integration with other photonic components on hybrid or monolithic platforms to realize fully functional all-optical processing circuits.

## Figures and Tables

**Table 1:** Key simulation parameters for the QD-SOA-MZI XNOR gate [5,11,12,13,61,62,63,64,65,66,67,68,72].

Symbol	Definition	Value	Unit
Optical Pulse Parameters			
E_0_	Pulse energy	0.2	pJ
τ_FWHM_	Pulse width (FWHM)	0.5	ps
T	Bit period	4	ps
N	PRBS length	127	-
Wavelength & Power			
λ_A_	Wavelength of data signals A	1580	nm
λ_B_	Wavelength of data signals B	1580	nm
λ_CW_	Wavelength of CW probe	1539	nm
P_A_	Power of data signals A	0.2	mW
P_B_	Power of data signals B	0.2	mW
P_CW_	Power of CW probe	0.4	mW
QD-SOA Electrical & Structure			
J	Injection current density	4	kA/cm^2^
d	WL thickness	0.5	μm
L	Active region length	1	mm
Γ	Optical confinement factor	0.15	-
QD-SOA Carrier & Gain			
N_ES_	Carrier density in QD ES	7.2 × 10^18^	cm^−3^
N_GS_	Carrier density in QD GS	3.6 × 10^18^	cm^−3^
a	Differential gain	8.6 × 10^−15^	cm^2^
G_0_	Unsaturated power gain	20	dB
E_sat_	Saturation energy	28	mW
QD-SOA Carrier Lifetimes			
τ_wd_	Transition rate from WL to QD state	5	ps
τ_dw_	Excitation rate from QD state to WL	1000	ps
τ_wr_	Carrier recombination rate in WL	2200	ps
τ_dr_	Carrier recombination rate in QD state	400	ps
QD-SOA Nonlinear Parameters			
τ_CH_	Temperature relaxation rate for CH	0.3	ps
τ_SHB_	Carrier-carrier scattering rate for SHB	0.1	ps
ε_CH_	Nonlinear gain suppression factor due to CH	0.02	W^−1^
ε_SHB_	Nonlinear gain suppression factor due to SHB	0.02	W^−1^
α	Traditional linewidth enhancement factor	5	-
α_CH_	Linewidth enhancement factor due to CH	1	-
α_SHB_	Linewidth enhancement factor due to SHB	0	-

**Table 2:** Comprehensive benchmarking of all-optical XNOR logic gates based on various semiconductor optical amplifier platforms, including conventional SOA, quantum-dot SOA (QD-SOA), photonic crystal SOA (PhC-SOA), reflective SOA (RSOA), and carrier reservoir SOA (CR-SOA) configurations. Performance metrics comprise operational data rate, quality factor (QF), and results type (experimental: Exp.; simulation: Sim.) with corresponding references.

Scheme	Results Type	Data Rate	QF	Reference
SOA				
SOA-OBPF	Exp.	40 Gb/s	-	[35]
SOAs-MZIs	Sim.	80 Gb/s	11.86	[72]
XPM (SOAs-MZIs)	Sim., Exp., Sim.	80 Gb/s	10.13	[74,75,76]
TPA (SOAs-MZIs)	Sim.	250 Gb/s	12.34	[77]
QD-SOA				
XPM (QD-SOAs-MZIs)	Sim.	160 Gb/s	29.72	[78]
TPA (QD-SOAs-MZIs)	Sim.	1 Tb/s	31	[79]
TPA (QD-SOAs-MZIs)	Sim.	2 Tb/s	9.8	[80]
PhC-SOA				
XPM (PhC-SOAs-MZIs)	Sim.	160 Gb/s	15.83	[81]
RSOA				
XPM (RSOAs-MZIs)	Sim.	160 Gb/s	15.83	[82]
CR-SOA				
CR-SOAs-MZIs	Sim.	120 Gb/s	12.40	[83]
TPA (CR-SOAs-MZIs)	Sim.	320 Gb/s	10.78	[84]
QD-SOA				
XPM (QD-SOAs-MZI)	Sim.	250 Gb/s	26.30	This Work

## Data Availability

The original contributions presented in this study are included in the article. Further inquiries can be directed to the corresponding authors.
